# G-Protein-Coupled Receptors in Rheumatoid Arthritis: Recent Insights into Mechanisms and Functional Roles

**DOI:** 10.3389/fimmu.2022.907733

**Published:** 2022-07-08

**Authors:** Jianan Zhao, Kai Wei, Ping Jiang, Cen Chang, Lingxia Xu, Linshuai Xu, Yiming Shi, Shicheng Guo, Dongyi He

**Affiliations:** ^1^ Guanghua Clinical Medical College, Shanghai University of Traditional Chinese Medicine, Shanghai, China; ^2^ Department of Rheumatology, Shanghai Guanghua Hospital, Shanghai University of Traditional Chinese Medicine, Shanghai, China; ^3^ Institute of Arthritis Research in Integrative Medicine, Shanghai Academy of Traditional Chinese Medicine, Shanghai, China; ^4^ Computation and Informatics in Biology and Medicine, University of Wisconsin-Madison, Madison, WI, United States; ^5^ Department of Medical Genetics, School of Medicine and Public Health, University of Wisconsin-Madison, Madison, WI, United States; ^6^ Arthritis Institute of Integrated Traditional and Western Medicine, Shanghai Chinese Medicine Research Institute, Shanghai, China

**Keywords:** G-protein-coupled receptors, rheumatoid arthritis, inflammation, bone destruction, lipid metabolism, angiogenesis

## Abstract

Rheumatoid arthritis (RA) is a chronic inflammatory disease that leads to joint damage and even disability. Although there are various clinical therapies for RA, some patients still have poor or no response. Thus, the development of new drug targets remains a high priority. In this review, we discuss the role of G-protein-coupled receptors (GPCRs), including chemokine receptors, melanocortin receptors, lipid metabolism-related receptors, adenosine receptors, and other inflammation-related receptors, on mechanisms of RA, such as inflammation, lipid metabolism, angiogenesis, and bone destruction. Additionally, we summarize the latest clinical trials on GPCR targeting to provide a theoretical basis and guidance for the development of innovative GPCR-based clinical drugs for RA.

## Introduction

Rheumatoid arthritis (RA) is an autoimmune disease characterized by synovial inflammation, joint destruction, and other clinical symptoms, including joint swelling, pain, morning stiffness, and weakness (1). The global prevalence of RA is approximately 0.5% to 1% (2). The risk factors for RA include genetic and metabolic aspects, genetic-environmental interactions, and microbial communities, all of which are involved in the pathogenesis of RA (3). For example, oxidative stress in multiple immune cells is thought to be an important factor in the chronic inflammatory destruction of RA, which in turn leads to joint destruction and extra-articular damage, including atherosclerosis, subcutaneous nodules and leg ulcers, systemic vasculitis, pulmonary fibrosis, scleritis and outer scleral inflammation, valvular heart disease and conduction abnormalities, and spinal cervical spondylosis (4). Currently, treatment options for RA include disease-modifying antirheumatic drugs (DMARDs), nonsteroidal anti-inflammatory drugs (NSAIDs), and biologics. Among them, analgesics and NSAIDs can reduce pain and stiffness, but NSAIDs have limited effectiveness and often have gastrointestinal and cardiac toxicity (5). DMARDs are the primary treatment and have been tried in combination, but some DMARDs have multiple adverse effects such as nausea, hepatotoxicity, hematometabolic disorders, and interstitial lung disease (5). Biological agents, including anti-tumor necrosis factor (TNF)-α antibodies, are also effective, but there are still adverse events, such as infusion and injection site infections, and differences in efficacy (5). With the advent of these new therapies, treatment of patients with RA has improved. However, due to heterogeneous factors and complex pathological mechanisms in RA, some patients have a poor clinical response, and the targeted development of new therapies is still a priority.

**Figure 1:**
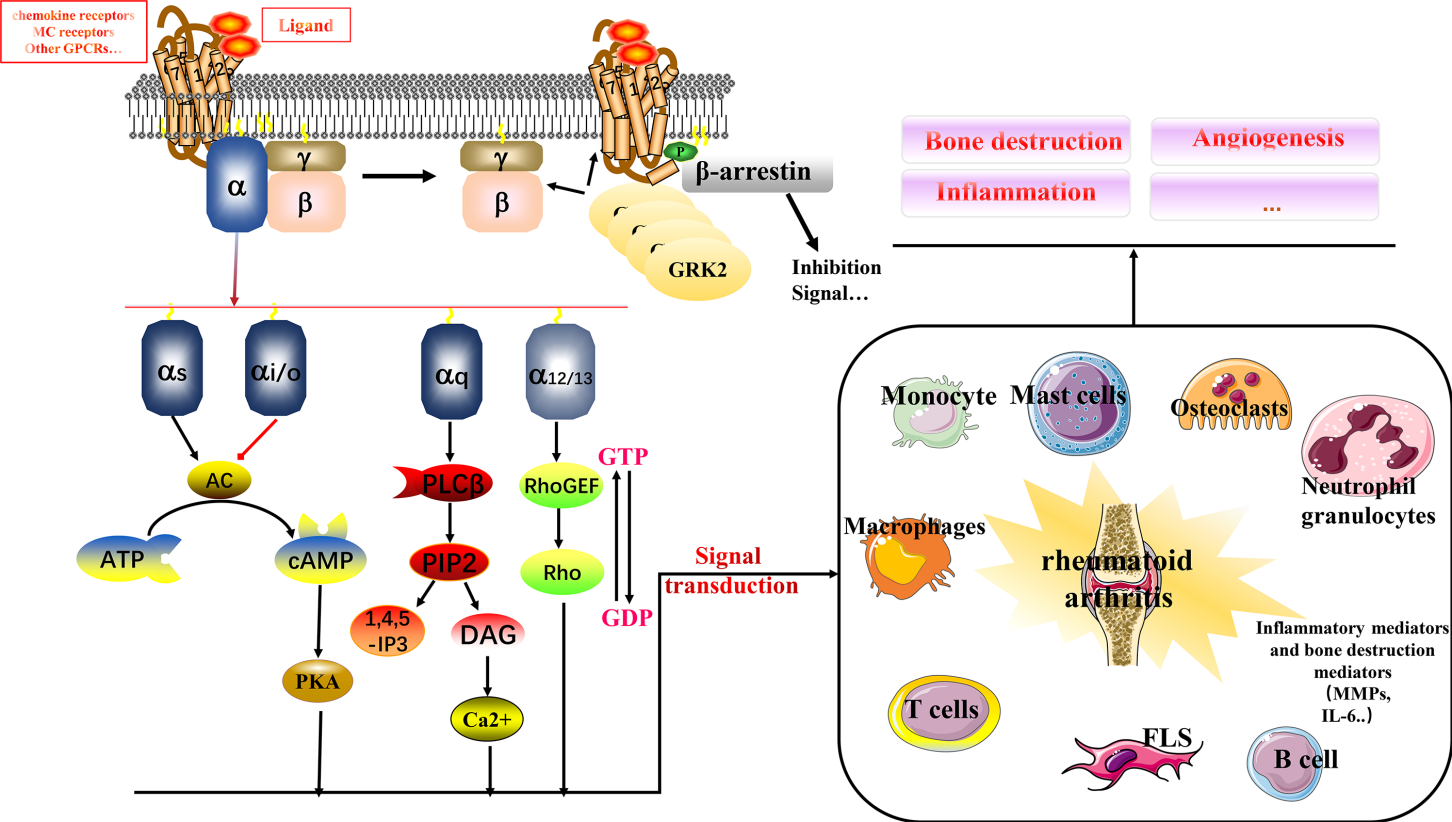
Relationship between multiple GPCRs and RA. GPCRs signal through the αβγ subunit of heterotrimeric G proteins. In response to ligand signals, GTP binds to the Gα subunit, and Gβγ dissociates to form a dimer for downstream signaling, binding to biological mediators. GRK2 is a kinase that phosphorylates activated GPCRs, making them a high-affinity substrate for the binding of the uncoupling protein arrestin. Arrestin binding abolishes (“arrests”) G protein-mediated signaling. The binding of GRK2 to Gβγ recruits cytosolic GRK2 to the plasma membrane, where the activated receptor is located. This is its primary function. It can also inhibit Gβγ-mediated signaling by sequestering Gβγ, which is a secondary effect. GPCRs interact with immune cells in RA to influence multiple mechanisms including bone destruction, inflammation, and angiogenesis.

G-protein-coupled receptors (GPCRs), also known as seven-transmembrane domain receptors, respond to signals such as hormones, neurotransmitters, odors, and light, and transmit signals to cells for physiological functions (6). GPCRs can be classified into glutamate, rhodopsin, adhesion, frizzled/taste2, and secretin families (7). The classical signaling process of GPCR has been well described and consists of three parts: receptor, G protein, and effector. G protein is a heterotrimer composed of α, β, and γ subunits (see **Figure 1** **)** (8). Briefly, in response to stimuli, the receptor’s structure begins to change to enhance binding to G proteins; the guanosine diphosphate (GDP) in the Gα subunit of resting G proteins is released; and is converted to guanosine triphosphate (GTP). The Gβγ dimer dissociates, which in turn activates downstream effectors to continue signaling, often accompanied by an increase in cyclic adenosine monophosphate (cAMP) and activation of protein kinase C (PKC). The specific downstream transmission signal depends on the α subunit species, including primarily Gαs, Gαi/o, Gαq/11, and Gα12/13 (6). GPCRs and their signaling pathways are involved in multiple human physiological and pathological processes. Drugs targeting GPCRs account for approximately 27% of the global drug therapy market (6). Multiple multifamily GPCRs in are linked to immune mechanisms in RA, including inflammatory responses. Therefore, in this review, we have investigated the mechanisms of GPCRs in RA. Based on our findings, GPCR-targeted drug development has an excellent potential and economic translational value for clinical therapy development in RA.

## Association of chemokine receptors and RA

Chemokine receptors are a class of GPCRs that regulate immunity. This class includes C-C motif chemokine receptor(CCR) 1-10, C-X-C motif chemokine receptors (CXCR) 1-7, X-C motif chemokine receptor 1 (XCR1), and C-X3-C motif chemokine receptor 1 (CX3CR1) (9). Chemokine receptors appear to be mostly Gαi-coupled, with some chemokine receptors also coupled to other G proteins, as verified by manual transfection in some contexts lacking Gαi/o proteins. Therefore, in the absence of specific information, chemokine receptors are by default considered Gαi/o-coupled (10). The overall chemokine and chemokine receptor cellular expression patterns in RA have been studied. For example, surface molecular assays of many peripheral blood B cells from RA patients revealed that CCR5, CCR6, CCR7, CXCR3, CXCR4, and CXCR5 play an essential role in B cell synovial migration, proliferation, and cytokine production (11). CCR1, CXCR4, and CCR5 are abundantly expressed in the RA synovium (12). Fibroblast-like synoviocytes (FLSs) in RA express CCR2, CCR5, CXCR3, and CXCR4. They have a pro-migration, proliferation, and matrix metalloproteinase production effect on FLSs under the stimulation of different ligands (13). Monocytes in synovial fluid mainly express CCR1, CCR2, CCR3, and CCR5, whereas peripheral blood expresses CCR1-5 at different levels of expression (14). Macrophages in rats with AIA mainly express CCR1, CCR2, and CCR5, which may maintain an inflammatory environment. CXCR4 is upregulated in endothelial cells and may be mainly associated with cell migration, angiogenesis, and inflammation (15). CCR1 and CXCR4 expression is upregulated in osteoblastic monocytes in RA (16). The roles of chemokines and chemokine receptors in RA have been previously reviewed (17–19). Therefore, we briefly summarize and update this information in the following sections (see **Tables 1** and **2**).

**Table 1:** Chemokine receptors and their ligands.

Chemokine receptor	Ligand	Cell expression in RA
CCR1	CCL3, CCL4, CCL5, CCL6, CCL7, CCL9, CCL15, CCL16, CCL23	T cell, NK cell, monocytes, macrophages, osteoblasts
CCR2	CCL2, CCL5, CCL7, CCL8, CCL12, CCL13, CCL16	FLS, B cell, monocytes, macrophages
CCR3	CCL4, CCL5, CCL7, CCL8, CCL15, CXCL10, CCL11, CCL13, CCL24, CCL26, CCL28	FLS, osteoclasts, monocytes, T cells (Th2), macrophages
CCR4	CCL2,CCL5,CCL3, CKLF1, CCL22, CCL17	FLS, T cell (Th2, Th17, Treg cell), monocytes
CCR5	CCL5, CCL3, CCL4, CCL8, CCL7,CCL14,CCL15	FLS, T cell (Th1), monocytes, macrophages
CCR6	CCL20	T cell (Th1 cell, Th22 cell, Th17.1 cell)
CCR7	CCL21, CCL19	Macrophages, monocytes, T cell
CCR9	CCL25	FLS, monocytes, macrophages
CCR10	CCL27, CCL28	Bone marrow cells, endothelial cells
CXCR1	CXCL5, CXCL6, CXCL8	FLS, monocytes, neutrophils
CXCR2	CXCL1, CXCL2, CXCL3, CXCL5, CXCL6, CXCL7, CXCL8	FLS, monocytes, neutrophils
CXCR3	CXCL4, CXCL4L1, CXCL9, CXCL10, CXCL11	FLS, plasma cells, mast cells, T cells (Th1)
CXCR4	CXCL12	FLS, T cells, monocytes, chondrocytes, endothelial cells, osteoblasts
CXCR5	CXCL13	T cell (Tfh, Tfr), B cell, endothelial progenitor cell
CXCR6	CXCL16	FLS, endothelial cells, T cell
CXCR7	CXCL12	Endothelial cells
CX3CR1	CX3CL1	NK cells, monocytes, CD4+ T and CD8+ T cells, osteoblasts
XCR1	XCL1, XCL2	Mononuclear cells

**Table 2:** Clinical trials of GPCR related to RA.

Name	Sponsor	ClinicalTrials.gov Identifier	Targets	Treatment	Condition or disease	Phase
TG-0054	GPCR Therapeutics, Inc.	NCT00822341	CXCR4	TG-0054	Healthy	Phase 1
POL6326	Polyphor Ltd.	NCT01841476	CXCR4	POL6326	Healthy	Phase 1
AZD4818	AstraZeneca	NCT00687232	CCR1	AZD4818, Placebo	Healthy	Phase 1
PF-04136309	Pfizer	NCT02598206	CCR2	PF-04136309	Healthy	Phase 1
RIST4721	Aristea Therapeutics, Inc.	NCT05023811	CXCR2	RIST4721	Healthy, ADME	Phase 1
RIST4721	Aristea Therapeutics, Inc.	NCT04105959	CXCR2	RIST4721, placebo	Inflammatory Response	Phase 1
AZD5069	AstraZeneca	NCT01332903	CXCR2	[14C] AZD5069	Healthy	Phase 1
	AstraZeneca	NCT00953888	CXCR2	AZD5069, placebo	Healthy	Phase 1
	AstraZeneca	NCT01100047	CXCR2	AZD5069, placebo	Healthy	Phase 1
	AstraZeneca	NCT01051505	CXCR2	AZD5069, placebo	Healthy	Phase 1
AZD2423	AstraZeneca	NCT00977626	CCR2	AZD2423, Placebo	Healthy	Phase 1
	AstraZeneca	NCT00940212	CCR2	AZD2423, Placebo	Healthy Volunteers	Phase 1
	AstraZeneca	NCT00970775	CCR2	AZD2423, Placebo	Healthy Volunteers	Phase 1
	AstraZeneca	NCT01233830	CCR2	AZD2423, Placebo	Healthy	Phase 1
PF-04634817	Pfizer	NCT01247883	CCR2, CCR5	PF-04634817	Healthy	Phase 1
	Pfizer	NCT01140672	CCR2, CCR5	PF-04634817	Healthy	Phase 1
	Pfizer	NCT01098877	CCR2, CCR5	PF-04634817, Placebo	Healthy	Phase 1
AZD5672	AstraZeneca	NCT00722956	CCR5	AZD5672, atorvastatin	Healthy Volunteers, Pharmacokinetics	Phase 1
	AstraZeneca	NCT00723424	CCR5	AZD5672, Digoxin	Healthy Volunteers, Pharmacokinetics	Phase 1
	AstraZeneca	NCT00746837	CCR5	AZD5672	Healthy Volunteers	Phase 1
	AstraZeneca	NCT00887770	CCR5	AZD5672, Moxifloxacin ,placebo	Rheumatoid Arthritis	Phase 1
	AstraZeneca	NCT00711074	CCR5	AZD5672	Rheumatoid Arthritis	Phase 1
	AstraZeneca	NCT00713544	CCR5	AZD5672, Etanercept , placebo	Rheumatoid Arthritis	Phase 2
	AstraZeneca	NCT00871767	CCR5	AZD5672	Rheumatoid Arthritis	Phase 1
Maraviroc	Pfizer	NCT00427934	CCR5	Maraviroc, placebo	Rheumatoid Arthritis,	Phase 2
NNC 0151-0000-0000	Novo Nordisk A/S	NCT02151409	C5aR	NNC 0151-0000-0000 ,placebo	Inflammation, Systemic Lupus Erythematosus, Rheumatoid Arthritis, Healthy	Phase 1
	Novo Nordisk A/S	NCT01223911	C5aR	NNC 0151-0000-0000 ,placebo	Inflammation, Rheumatoid Arthritis	Phase 2
NNC0215-0384	Novo Nordisk A/S	NCT01955603	C5aR	NNC0215-0384 ,placebo	Inflammation, Rheumatoid Arthritis	Phase 1
	Novo Nordisk A/S	NCT01611688	C5aR	NNC0215-0384 ,placebo	Inflammation, Rheumatoid Arthritis	Phase 1
CCX354-C	ChemoCentryx	NCT01027728	CCR1	CCX 354-C	Rheumatoid Arthritis	Phase 1 Phase 2
	ChemoCentryx	NCT01242917	CCR1	CCX 354-C, placebo	Rheumatoid Arthritis	Phase 2
PF06835375	Pfizer	NCT03334851	CXCR5	PF06835375, placebo	Systemic Lupus Erythematosus, Rheumatoid Arthritis	Phase 1

### CCR1, CCR2, and CCR5

CCR1 is coupled with Gαi/o (20). CCL3 (MIP-1α), CCL4, CCL5 (RANTES), CCL6 (MIP-related protein-1), CCL7, CCL8, CCL9 (MIP-1γ/MIP-related protein-2), CCL15 (MIP-1δ/hemofiltrate CC chemokine-2/leukotactin-1), CCL16, and CCL23/CKβ8/myeloid progenitor inhibitory factor-1) have been described as CCR1 ligands (21, 22). The number of peripheral blood CCR1+ T/NK lymphocytes in patients with RA is negatively correlated with IL-10, whereas the number of CCR2+ B cells is positively correlated with IL-6 (23). The expression of CCR1 was positively correlated with serum antibodies against anti-cyclic citrullinated peptide (anti-CCP) in patients with RA (24). Several preclinical experimental studies have shown that CCR1 inhibition improves arthritic symptoms. For example, CCR1 inhibition reduces inflammation, joint damage, and cellular infiltration in the collagen-induced arthritis (CIA) mouse model. The specific mechanism may involve reduced recruitment of inflammatory cells; however, it is noteworthy that CCR1 inhibition spontaneously increases TNF-α levels in certain settings (25). The favorable animal results for CCR1 further support the clinical development of CCR1 antagonists. A phase IIa, double-blind, placebo-controlled, randomized, proof-of-concept study investigated the efficacy of a CCR1 antagonist (MLN3897) in RA patients; MLN3897 was well tolerated, but the results were not favorable, and there was no significant difference in its efficacy (26). Clinical blockade of CCR1 is likely to be effective and may always require maintenance of high levels of receptor occupancy (27). The CCR1 antagonist (CCX354-C) has shown initial clinical effectiveness. In a randomized placebo-controlled clinical trial in patients with RA, a CCR1 antagonist (CCX354-C) demonstrated good safety, tolerability, and clinical activity. CCX354 showed good tolerability and a linear dose-exposure profile in healthy subjects (28, 29).

The ligands of CCR2 include CCL2, CCL5, CCL7, CCL8, CCL12, CCL13, and CCL16 (22, 30, 31). In RA, the human isoforms of CCR2 include CCR2a and CCR2b, which are coupled to Gαi/o in most cases and Gαq in some cases and trigger the canonical activation of phospholipase Cβ isoenzymes downstream (32, 33). The mRNA expression of CCR2 was significantly higher in the range of 2.6< the disease activity score-28 (DAS28) <5.1 than DAS28>5.1 and control. CCL2 was negatively correlated with DAS28 index. Thus, CCR2 may contribute to early and rapid progression of inflammation (24). For example, the binding of CCR2 and CCL2 can help monocytes migrate to sites of RA inflammation and differentiate into M1 proinflammatory macrophages, possibly linking RA inflammation to insulin resistance (34). However, a double-blind, randomized, placebo-controlled clinical trial investigating the efficacy of a CCR2-blocking antibody (MLN1202) in patients with RA found that CCR2 blockade was not sufficient to improve multiple symptoms of RA (35).

CCR5 couples with Gαi/o and Gαq, and its ligands include CCL5, CCL3, CCL4, CCL8, CCL7,CCL14, and CCL15 (22, 31, 36, 37). CCR5 expression in rheumatoid factor (RF)-negative patients with RA is markedly higher than that in RF-positive patients with RA, which may be a molecular basis for the differences in RF expression in patients with RA (24). Investigators have found that the number of CCR5 molecules expressed on the cell surface correlates with the intensity of tumor necrosis factor α (TNFα) -induced T cell migration to the joint in RA. Anti-CCR5 antibodies can block this migration effect, thus possibly linking it with joint inflammation (38). Similar to the failed clinical trial of CCR2, clinical trials of CCR5 antagonists suggest that silencing CCR5 alone is not a truly effective target for RA (39–41). In conclusion, the lack of clinical efficacy of CCR2 and CCR5 blockade may be because their role in the migration of monocytes to the synovial membrane in patients with RA is not critical.

### CCR3

CCR3 may be coupled with Gαi/o (42). The ligands of CCR3 are CCL4, CCL5 (RANTES), CCL7 (MCP-3), CCL8 (MCP-2), CCL15 (HCC-1), CXCL10 (interferon (IFN)-γ inducible protein-10), CCL11 (eotaxin), CCL13, CCL24 (eotaxin-2), and CCL26 (eotaxin-3) (17, 22, 31). The role of CCR3 in RA may include the induction of cell migration and promotion of bone destruction and act as a predictor of the efficacy of certain clinical therapies. For example, flow cytometry analysis of synovial cells from patients with RA revealed that most CCR3+ cells were FLSs and that CCL11 expression was upregulated in the plasma and synovial fluid (43). IL-1β enhances the release of CCL11 from FLSs; CCL11 induces upregulation of CCR3 and matrix metalloproteinase (MMP)-9 mRNA expression in FLSs (43) and can induce the migration of FLSs and monocytes (44). CCL11 is expressed in osteoblasts and its expression is enhanced in response to inflammatory stimuli. Osteoclasts expressing CCR3 interact with exogenous CCL11 to stimulate cell migration and bone resorption (45). CXCL10, with an increased expression in synovial cells of patients with RA in response to TNF-α stimulation, interacts with CCR3 in T-cells and mediates an increase in receptor activation of nuclear factor kappa-B ligand (RANKL) expression *via* the Gαi subunit. As a result, osteoclast genesis and bone destruction are enhanced (46).Reduced CCR3 expression in serum CD4+ lymphocytes and reduced number of synovial CCR3+ monocytes in RA patients treated with steroids and anti-TNFα (47). In contrast, CD4 T cells and CD14 monocytes expressing CCR3 and CD8 T cells expressing CCR5 were increased in the peripheral blood of RA patients treated with anti-TNFα antibodies. This suggests the restoration of peripheral cell-mediated immune function, thereby blocking aggregation in the joints and inhibiting inflammation (48). The role of other CCR3 ligands in RA has been well studied and summarized (17–19). For example, CCL5 is mainly involved in the migration of leukocytes, and the use of anti-CCL5 antibodies can reduce the pathological manifestations of arthritis in the CIA mouse model (49). CCL24 is a major chemokine for inflammatory cells, and adjuvant arthritic mice treated with anti-CCL24 antibody showed significantly improved arthritic symptoms and inflammatory responses (50). Clinical inhibitors of CCR3 are in development, but currently do not target RA directly; most inhibitors target other diseases to test their effects, such as Parkinson’s disease, macular degeneration, and diabetic retinopathy.

### CCR4, CCR6, and CXCR3

The ligands of CCR4 include chemokine-like factor 1 (CKLF1), CCL2, CCL3, CCL5, CCL17, and CCL22 (22, 31, 51, 52). CKLF1 is significantly positively correlated with C-reactive protein (CRP) and erythrocyte sedimentation rate (ESR) (51). The expression of FLS in CCR4 may promote cell migration and proliferation (53). CCR4 expression in pro-inflammatory T-cell populations may promote inflammation by facilitating cell migration process. For example, a significant increase in circulating CCR4+ CXCR3-helper T cells (Th2 and Th17 cells) has been observed in clinically untreated patients with early RA (54). In addition, the expression of Treg cells of CCR4 may be a regulatory negative feedback mechanism of inflammation. For example, CCR4 and CCR6 expression was upregulated in peripheral blood Treg cells from patients with active RA and it was positively correlated with DAS28, suggesting that they could migrate to joints (55). CCR4+ T cells exert anti-inflammatory effects in patients with juvenile rheumatoid arthritis (JRA) by producing anti-inflammatory cytokines (IL-4) (56). However, this is not sufficient for the suppression of inflammation, so there are many proinflammatory mechanisms, such as CCL22, which is increased in both synovial fluid and serum of patients with RA, and may bind Treg cells CCR4 suppresses the number of Treg cells through the signal transducer and activator of transcription 5 (STAT5) signaling pathway (57).

CCL20 is the ligand of CCR6 (31). CCR6 expression may be primarily associated with T-cell subsets in RA. The proportion of CCR6+ memory T helper cell populations in anti-cyclic citrullinated peptide antibody (ACPA)+ versus ACPA- patients with RA is significantly different. This suggests that other cell subpopulations may be involved in various mechanisms of RA (58). The CCR6+ memory T helper cell population contains Th1/Th22 and Th17.1 cells, which activate FLSs in an IFN-γ-independent manner (59). In response to TNF-α, interleukin (IL)-6, and IL-1β stimulation, Th22 cells expressing CCR4, CCR6, and CCR10 migrate to the synovial tissues of patients with active RA and produce IL-22 to stimulate osteoclast differentiation for bone destruction (60). T cells and synovial cells in RA synovium produce CCL20, which binds to CCR6. High expression of RAR-related orphan nuclear receptor (ROR) γt promotes CCR6 expression, enhancing Th17 cell migration into the joint and promoting inflammation (61, 62).

The ligands of CXCR3 are CXCL4, CXCL4L1, CXCL9, CXCL10, and CXCL11 (22, 31). CXCL10-induced cell migration was found to require CXCR3, EGFR, and Gβγ subunits downstream of CXCR3, but not Gαi/o (63). CXCR3 expression in plasma cells, mast cells, and T-cell subsets is associated with RA. Plasma cells from patients with RA express CXCR3 by interacting with sub-synovial FLS-expressing Mig/CXCL9 recruited to the subsynovial lamina (64). In addition, mast cells in RA synovial tissue abundantly express CXCR3; this may maintain the synovial inflammatory environment by binding CXCL9 and CXCL10 and producing mediators, including histamine, proteases, arachidonic acid metabolites, and cytokines (65). Patients with RA appear to express CCR5 and CXCR3 preferentially on Th1 cells, and Th2 cells preferentially express CCR3, CCR4, and CCR8 (66, 67). The combination of MTX and anti-TNFα antibodies decreased CXCR3 and IL-12R expression (considered a Th1 cell markers) and upregulated CCR4 and IL-4R expression (considered a Th2 cell markers) in peripheral blood CD4 cells in patients with RA, with a high percentage of apoptotic cells (68). Paradoxically, peripheral blood CD4 and CD8 CXCR3+ T lymphocytes were increased in RA patients treated with infliximab and etanercept. CXCR3+ CD4 T lymphocytes negatively correlated with DAS28 (69). This seems to validate the findings of Nanki et al. that chemokine receptor expression did not differ significantly across T-cell subpopulations (70). Further experiments are needed, but restoring the suppression of inflammation by regulating the balance between Th1/2 cells is a feasible strategy to improve RA. Several preclinical trials have demonstrated that targeted inhibition of CXCR3 is beneficial for the treatment of RA. Specifically, it reduced the recruitment of Th1 cells to sites of inflammation (71) and restored the balance between Th17 and Treg cells (72).

### CCR7

CCR7 may be coupled with Gαi/o and Gαq (73). CCR7 ligands, including CCL21 and CCL19, are associated with the homing and localization of dendritic cells and T cells (74). CCR7 is a surface marker of macrophages in the RA synovial fluid. Its upregulation in cells may promote inflammation and is positively correlated with DAS28 and inflammatory factor levels, and negatively correlated with anti-inflammatory factor levels in patients with RA. For example, CCR7+CD95+CD4+ peripheral lymphocytes were significantly elevated in patients with active RA and were positively correlated with IL-6 (75). Lipopolysaccharides (LPS) and IFN-γ promote CCR7 expression. IL-4 inhibits CCR7 expression. CCL21 promotes Th17 differentiation, osteoclast formation, angiogenesis, and proinflammatory macrophage differentiation (76, 77). In addition, miR-155 expression was higher in ACPA+ patients than in ACPA − patients and was correlated with DAS28. miR-155 can promote CCR7 expression and downregulate CCR2 expression in RA monocytes (78). The progression of arthritis in CIA mice can be inhibited using an anti-CCR7 antibody (74). The development of clinical inhibitors of CCR7 for non-Hodgkin’s lymphoma and chronic lymphocytic leukemia is currently underway.

### CCR9, CCR10

The association of CCR9 and CCR10 with RA is less direct, with CCR9 associated primarily with cell migration of FLS, proinflammatory differentiation of macrophages, and related inflammatory and bone destruction processes. CCR10 is primarily associated with angiogenesis. CCL25 is the ligand of CCR9. In response to TNF-α, CCR9 elevated expression in peripheral blood monocytes and macrophages of RA and in combination with CCL25 induces cell migration (79) and differentiation of monocytes into M1 macrophages *via* the P38 and extracellular signal-regulated kinase (ERK) pathways (80). FLSs and macrophages release CCL25 in RA synovial fluid in response to stimulation by IL-1β and IL-6. CCL25 binds to CCR9 on FLSs and macrophages to promote osteoclast formation and vascular opacification (80). Additionally, it stimulates IL-6 and MMP-3 production in FLSs and IL-6 and TNF-α production in peripheral blood mononuclear cells. The antagonism of CCR9 suppressed arthritic symptoms in the CIA mouse model (81).

The ligands of CCR10 are CCL27 and CCL28 (82). CCR10 and ligand CCL28 expression is elevated in the synovial tissue and synovial fluid of patients with RA, mainly in the bone marrow and endothelial cells; inhibition of CCL28 or CCR10 reduces endothelial cell migration and angiogenesis (83). Crohn’s disease is a focus in the development of clinical inhibitors for CCR9 and a gap exists in clinical inhibitors for CCR10. Therefore, further clinical trials of both for RA are still necessary.

### CXCR1, CXCR2

Both CXCR1 and CXCR2 are coupled to Gαi/o proteins (22, 84). The ligands of CXCR1 include CXCL5, CXCL6, and CXCL8. The ligands of CXCR2 include CXCL1, CXCL2, CXCL3, CXCL5, CXCL6, CXCL7, and CXCL8, and interact with GRK6 to negatively regulate receptor sensitization and transport, thereby affecting cell signaling and angiogenesis (22, 84). CXCR1 and CXCR2 are involved in the migration of neutrophils, FLS, T-cells, and monocytes in RA, influencing the subsequent inflammation and bone destruction processes and are expressed in their cell populations (85, 86). For example, CXCR1 and CXCR2 ligands, such as CXCL1, CXCL5, and LTB4, are highly expressed in the joints and bind to CXCR1 and CXCR2. Here, they promote joint migration of neutrophils in AIA mice (87). Targeted inhibition of neutrophil migration by the CXCR1 and CXCR2 antagonists SCH563705 reduces disease activity scores, joint inflammation, and bone and joint destruction in CIA mice (88). In addition, CXCL5 [epithelial neutrophil-activating peptide 78 (ENA-78)] of citrulline is significantly elevated in the synovial fluid of RA patients. It correlates with CRP and ESR, inducing inflammatory cell focus and inflammation through CXCR1-and CXCR2-induced chemotaxis of monocytes (89). Reduced CXCR1 and CCR2 expression in blood T cells after treating patients with RA with the anti-TNF-α antibody infliximab may inhibit their migration to sites of inflammation (90).

### CXCR4

CXCR4 may be coupled to Gαi/o and Gαq (73). The ligand of CXCR4 is mainly CXCL12 [stromal cell-derived factor 1 (SDF-1)]. High expression of CXCL12 and CXCR4 in the serum and synovial fluid of patients with RA is positively correlated with ESR, CRP, RF, and DAS28 scores (91). T-cell expression of CXCR4 may promote cell migration and inflammatory processes. CXCR4 is positively correlated with memory-activated CD4+ T cells and follicular helper T (Tfh) cells (92).Pablos et al. found that FLSs specifically express CXCL12, which aggregates and fixes in the heparan sulfate molecules of vascular endothelial cells, promoting angiogenesis and inflammatory cell infiltration. CXCL12 activates CXCR4 on the surface of T cells. Simultaneously, very late activation antigen 4 (VLA-4) interacts with vascular cell adhesion molecule 1 (VCAM-1) to promote the recruitment of T cells and their retention in the joints to promote inflammation (93, 94). The expression of CXCR4 in B cells may be indicative of disease activity; for example, considerable infiltration of P-gp+CXCR4+ CD19+ B cells can be observed in patients with RA, which may correlate with disease activity, drug resistance, and progressive joint destruction (95).

In addition, monocyte expression of CXCR4 may promote the process of cell migration and differentiation into proinflammatory macrophages, which can affect subsequent inflammatory and bone destruction processes. The hypoxic microenvironment induces SDF-1 expression in RA FLSs, leading to the accumulation of CXCR4-expressing monocytes in the synovial membrane and their differentiation into macrophages and secretion of proinflammatory factors (IL-1β, IL-6, and TNFα) and MMP. This process mediates inflammation and osteoarthritic destruction (96). The specific mechanism may involve SDF-1α enhancing the binding of c-Jun to AP-1 on the IL-6 promoter and enhancing AP-1 transcriptional activity to regulate IL-6 expression (97). Similarly, SDF-1α activates ERK/c-Fos/c-Jun *via* CXCR4 to mediate AP-1 activation of MMP13 to promote cartilage destruction (98). SDF-1a also stimulates CXCR4+ chondrocytes to release MMP3, which destroys bone joints (99).In addition, the endogenously released reduced form of HMGB forms a heterotrimeric complex with CXCL12; this complex interacts with CXCR4 to promote cell migration and inflammation *via* the toll-like receptor (TLR) 2/4 (100).

Therefore, the inhibition of CXCR4 is beneficial for improving RA. The herbal compound QLY can reduce joint lesions and paw swelling in adjuvant arthritis rats by a mechanism involving the inhibition of the CXCL12/CXCR4/nuclear factor kappa-light-chain-enhancer of activated B cells (NF-kB) pathway (101). In addition, targeted CXCR4 inhibitors are now well developed, and described in many excellent reviews (102–105). Many small-molecule compounds have significant CXCR4 inhibitory effects. We have focused on the progress of CXCR4 inhibitor development in clinical trials and have summarized and updated them here. The CXCR4 antagonist AMD3100 has been approved by the FDA for use as a hematopoietic stem cell mobilizer. All information was obtained from www.clinicaltrials.gov (See **Table 1**).

### CXCR5, CXCR6, and CXCR7

CXCL13 is the ligand of CXCR5. Changes in CXCR5 expression in different T-cell subsets may be associated with RA. The circulating Tfr/Tfh cell ratio in RA is negatively correlated with serum CRP, ESR, RF, anti-CCP, IgG, and the DAS28 index (106). Tfh cells can activate B cells and produce specific antibodies, and the number of Tfh cells in peripheral blood is positively correlated with anti-CCP antibody levels (107). In contrast, in RA remission, circulating Tfr cell subsets increase and are negatively correlated with RF, anti-cyclic citrullinated peptide, and DAS28 (108). A decrease in CD4+CXCR5+Foxp3+ Tfr cells/CD4+CXCR5+ Tfh cells in the peripheral blood of patients with RA may contribute to multiple humoral immune mechanisms (109). *In vitro* co-culture of RA FLSs and peripheral blood mononuclear cells (PBMCs) revealed that increased production of TNF-α, IL-1β, IL-6, and reactive oxygen species (ROS) could promote CD4 + CXCR5 + ICOS + Tfh differentiation (110). CXCR5 expression in other cells (endothelial progenitor cells and regulatory B cells) has also been linked to RA. CXCL13 expression is elevated in the synovial fluid of CIA mice and patients with RA. CXCL13 interacts with CXCR5 in endothelial progenitor cells and promotes homing and angiogenesis through the PLC, MEK, and AP-1 signaling pathways (111). CXCR5 expression on the surface of regulatory B cells (Bregs) is downregulated. The response to CXCL13 is not sufficient for preferential migration to the synovial fluid to produce sufficient anti-inflammatory factor IL-10, which may be one of the mechanisms of RA inflammation (112). CXCR5-deficient mice with impaired germinal response centers are resistant to collagen-induced arthritis (113). The application of clinical inhibitors of CXCR5 in RA is ongoing, and promising results are expected to be published.

The ligand of CXCR6 is CXCL16. CXCR6 is expressed in FLS, endothelial cells, and T-cells in RA. CXCR6 and CXCL16 levels are elevated in both RA FLSs and may stimulate FLS proliferation (114). CXCL16 also stimulates RANKL expression in RA FLSs through Janus kinase (JAK)2/STAT3 and P38/mitogen-activated protein kinase (MAPK) signaling (115). CXCR6 is also expressed in endothelial cells and may be involved in endothelial cell recruitment and angiogenesis in the RA joints by binding to CXCL16 (116). CXCR6 and CXCL16 can promote inflammation by affecting T cell differentiation and homing to joints. CXCR6 knockout CIA mice exhibit multiple arthritic symptoms (117, 118).

The ligand of CXCR7 is CXCL12 and was found to be internalized and signaled downstream in a chemokine-dependent manner only through recruitment of the β-arrestin protein (119, 120). CXCR7 binds to chemokines with a high affinity, but ligand binding does not lead to G protein-mediated intracellular calcium mobilization or chemotaxis (121). CXCR7 is expressed on endothelial cells in the synovium of RA patients and can be significantly upregulated in response to IL-1β and CXCL12 stimulation. It promotes angiogenesis, and is mainly used as an alternative receptor for CXCR4. CXCR7 inhibitors significantly reduced arthritic symptoms and vascularity in CIA mice (122).

### CX3CR1 and XCR1

CX3CR1 is mainly expressed in NK cells, monocytes, and CD4+ and CD8+ T cells. Its ligand CX3CL1/FKN (fractalkine) is primarily involved in cell adhesion (123). CX3CR1/CX3CL1 are also associated with the production of inflammatory mediators by macrophages, T cells, and FLSs in RA (124). CX3CR1+HLA-DRhiCD11c+CD80-CD86+ cells, an osteoclast subpopulation, are present in the synovium of patients with RA. In mice, the corresponding subpopulation CX3CR1hiLy6CintF4/80+I-A+/I-E+ cells can be inhibited by inhibiting FOXM1 (125). Patients with active RA treated with infliximab and etanercept had reduced CX3CR1 expression in peripheral blood PBMC and T cells and serum CX3CL1 levels (126, 127).

The ligands of XCR1 are XCL1 and XCL2 (22). XCR1 expression is upregulated in mononuclear cells (MNCs) of synovial fluid and venous blood samples from patients with RA, suggesting that XCR1 may be involved in the mechanism of RA (128). The connection between CX3CR1 or XCR1 and RA has not been well studied, and the specific mechanisms require further study.

## Activation of melanocortin (MC) receptor improves RA

The structure and function of MC and its receptors have been well-reviewed (129). MCR mainly included MC1-5R. MC1-5R is coupled with Gαs to stimulate downstream cAMP/PKA signaling. In addition, M3CR promotes downstream ERK1/2 signaling to promote cell proliferation by coupling with Gαi/o. MC4R also promotes downstream protein kinase C by coupling with Gαq and inhibits apoptosis by coupling with Gαi to promote downstream ERK1/2 signaling. MC5R can also couple Gαi/o to promote downstream PI3K/C-Raf/MEK1/2/ERK1/2 signaling (130). Activation of MCR may have an ameliorative effect on RA. MC3R knockdown with serum transfer-induced arthritis exacerbated significant bone erosion, high expression of RANKL in the joint, increased time of NF-kB activation, and upregulation of proinflammatory genes [IL-1β, IL-6, and nitric oxide synthase 2 (NOS2)]. Conversely, overexpression of MC3R and MC3R agonist D [Trp8]-γ-MSH significantly reduced the degree of arthritis, suggesting that MC3R may be an important target in preventing bone destruction and inflammation progression in arthritis (131). Endogenous MCR agonists include ACTH and MSH (α, β, and γ), with different affinities for the five MCRs, where MC2R was activated only in response to ACTH (130).

Alpha-melanocyte-stimulating hormone (α-MSH) is a 13-amino acid peptide (132). α-MSH can inhibit inflammation by interacting with the MCR of multiple cells in RA *via* multiple mechanisms (132). Elevation of α-MSH may be a measure of suppression of inflammation in joint tissues. Catania et al. observed significantly elevated synovial fluid α-MSH, interleukin 1 receptor antagonist (IL-1ra), and soluble tumor necrosis factor receptor (sTNFr) levels in patients with RA (133). *In vitro*, α-MSH inhibits LPS-induced protein hydrolase release from macrophages, oxidative burst response, production of reactive oxygen and reactive nitrogen species, adhesion molecule expression, NF-kB activation, and downregulation of cell surface CD14 expression to inhibit inflammation (134). The inhibitory effect of NF-kB may be mediated by an increase in the cAMP-mediated activity (135). Similarly, Bhardwaj et al. found that the C-terminal tripeptide of α-MSH induces the production of the anti-inflammatory factor IL-10 in monocytes (136). α-MSH stimulation of T cells *in vitro* promotes phenotypic conversion of T cells to CD25+CD4+ regulatory T cells. It induces TGF-β production to suppress IFN-γ production by other effector T cells; eventually, inflammation is suppressed *via* a mechanism involving α-MSH binding to MC5R on T cells (137). Thus, synthetic MCR agonists may also inhibit inflammation in patients with RA. For example, Montero-Melendez et al. found that the pro-senescence effect of FLSs *via* agonist-induced MC1R expression suppressed the inflammatory response in RA (138). The MC1R agonist BMS interferes with FLSs cell cycle, anti-inflammatory, and arthroprotective features These include cell proliferation, cycle arrest, lysosomal amplification, expression of the cellular senescence marker p16 INK4, downregulation of cell cycle promoters and anti-apoptotic signals, downregulation of proinflammatory factors (CCL2, IL6, and IL8), increased expression of MMPs, and downregulation of matrix-degrading enzymes (ADAMTS1 and ADAMTS2) (138). Montero-Melendez et al. also found that MC Pan Agonist AP214 reduced disease scores and paw edema (which primarily affects IL-1β release) in a K/BxN serum transfer arthritis model, exerting an anti-inflammatory effect (139).

α-MSH is also linked to RA and is osteoprotective. α-MSH acts directly on the bone, increases bone turnover, and reduces bone volume, possibly through the synergistic action of other cells such as adipocytes or islet B cells (132). Human chondrocytes express a variety of MCRs such as MC2R and MC5R. *In vitro*, a-MSH stimulation can regulate cAMP, proinflammatory factors, and MMP in chondrocytes, which may play a role in inflammation, development, and cartilage degeneration (140). Similarly, Kaneva et al. found that α-MSH and D[Trp8]-γ-MSH inhibited TNF-α-induced proinflammatory factor release (IL-1, IL-6, and IL-8) from C-20/A4 chondrocytes, increased anti-inflammatory factor IL-10 release, decreased the expression of *MMP1, MMP3*, and *MMP13*, and inhibited the apoptosis of key molecules caspase3/7 and cell death. MC3R/4R antagonists inhibited the effects of D[Trp8]-γ-MSH, suggesting that MC1R and MC3R may be the primary MCRs for these mechanisms (141). Zaidi et al. found that ATCH induced vascular endothelial growth factor (VEGF) secretion *via* MC2R and stimulated osteoblast maturation and survival. This reduced experimental osteonecrosis induced by methylprednisolone acetate, which may be a mechanism to inhibit bone destruction in RA (142).

## Other GPCRs associated with RA

In addition to the intensively studied MCRs and chemokine receptors described above, there are also some GPCRs that are associated with RA For example, GPR120 regulates lipid metabolism and GPCR (CD97) is related to adhesion. Further experiments are needed to investigate their specific roles in depth.

### C5aR and Adenosine Receptors

C5aR is Gαi/o-coupled (143). C5aR is mainly expressed in neutrophils and macrophages. Elevated C5aR and C5a levels have been found in the blood and synovial fluid of RA patients (144). Patient mast cells also express C5aR and release histamine to participate in the inflammatory response (145). In the presence of multiple cytokines, neutrophils express chemokine receptors in the joint to release LTB4 and IL-1β to promote their recruitment. Simultaneously, immune complexes in RA can activate C5a production by neutrophils and bind C5aR to further amplify the inflammatory response (146). In addition, the plasma kallikrein-kinin system (KKS) is present in RA. KKS activation activates prekallikrein (pKal) and factor XII (FXII) cleavage of high-MW kininogen (HK) to release bradykinin. Kal also activates monocytes to promote proinflammatory cytokines, upregulate C5aR and FcRIII expression, and release C5a. Inhibition of KKS alleviates symptoms in arthritic mice (147). Macrophage infiltration in zymosan-induced arthritis (ZIA) mice; increased expression of C5aR and C3aR in joints; and elevated levels of C5a and soluble receptor activator of nuclear factor kappa B ligand (sRANKL) in synovial fluid are possible mechanisms of inflammation (148). Targeted inhibition of C5aR remains challenging in terms of clinical translation, although preclinical data have shown that anti-C5aR antibodies can improve symptoms in experimental arthritic mice (149). However, a double-blind placebo-controlled study found no clinical improvement in patients with RA receiving a C5aR inhibitor (150).

Adenosine is an anti-inflammatory mediator that acts mainly through four receptors (A1, A2A, A2B, and A3) to inhibit proinflammatory factors (IL-6 and TNF-α) and to promote the synthesis of anti-inflammatory factors (IL-10) (151). Its role in RA has been extensively reviewed and is mainly related to the mechanism of action of methotrexate, phosphoinositide 3-kinases (PI3Ks)/protein kinase B (PKB), and NF-kB signaling pathways. The detailed mechanism of action has previously been explained (152–156).

### GPR120

GPR120 is Gαi/o- or Gαq-coupled, and its ligands are LCFAs, unsaturated fatty acids, omega-3 fatty acids, and omega-6 fatty acids (157). Abnormalities in lipid metabolism were observed in CIA mice. For example, the antioxidant enzymes LDH and lipoproteins are positively correlated with the lipid fractions in the plasma and joint tissue. Lipid content is negatively correlated with lipid levels, and cytokine content is correlated with lipid fractions and the saturated fatty acid/unsaturated fatty acid ratio (158). Therefore, in addition to the effects of multiple immune mechanisms on RA, lipid metabolism may also regulate RA. GPCRs have been shown to regulate lipid metabolism, which may have potential therapeutic effects on RA. Some endogenous n-6 polyunsaturated fatty acids (PUFA) can cause inflammation and pain by promoting the synthesis of leukotrienes, prostaglandins, IL-6, TNF-α, and ROS. Simultaneously, exogenous linolenic acid, an n-3 PUFA that activates FFA4 (GPR120) and has potent anti-inflammatory effects, has been shown to improve inflammation and disease severity in RA (159, 160). Omega-3 fatty acids activate GRP120, which decreases transforming growth factor-β activated kinase 1 (TAK1) and inhibits the downstream IKKβ/I-κB pathway and terminal NF-κB. Thereby it suppresses T cell activation and inflammatory responses, and improves symptoms in arthritic mice (161). GPR120 inhibits CD40L-induced activation of dendritic cells and proinflammatory responses, and GPR120 agonists have similar effects (162).

### GPR43

GPR43 is Gαi/o- or Gαq-coupled, and its ligands are acetate, propionate, and butyrate (157). GRP43/FFA2R is expressed on RA FLSs and upregulated in response to TNFα stimulation, and its inhibition of GPR43 can significantly inhibit a variety of biological mediators and signaling pathways in RA, including IL-6, IL-8, high mobility group protein 1 (HMG-1), monocyte chemoattractant protein 1 (MCP-1), intercellular adhesion molecule 1 (ICAM-1), and vascular cellular adhesion molecule 1 (VCAM-1), production of ROS and 4-hydroxynoneal, MMP-3, and MMP-13, and activation of the NF-κB inflammatory signaling pathway (163).

### GPR91

GPR91 is Gαi/o- or Gαq-coupled, and its ligands is succinate (157). GPR91 is mainly expressed in macrophages and dendritic cells. GPR91 in dendritic cells triggers intracellular calcium flow by binding succinate; induces cell migration; and interacts with TLR to release proinflammatory factors. Succinate also enhances T helper cell activation (164). Succinate is abundant in the RA synovial fluid. Macrophage sensing of endogenous LPS activates TLR, releases succinate, and upregulates GPR91 sensing of succinate to activate glycolytic pathways and promote IL-1β production. GPR91 knockout mice have reduced macrophage activation and IL-1β release in response to antigen-induced arthritis (165). Intracellular succinate promotes VEGF production by activating GPR91 and inducing angiogenesis *via* HIF-1α (166).

### CD97

CD97 is primarily coupled to the Gαi/o protein (167). CD97 plays multiple roles in RA through its N-terminal epidermal growth factor structural domain combined with the N-terminal short consensus repeat structural domain of CD55 (168). For example, CD97 is expressed in granulocytes and monocytes, and is upregulated in T and B cells combined with CD55 activation to promote T cell activation (169). Neutralizing anti-CD97 antibody attenuates multiple arthritic manifestations in a collagen-induced murine joint model (170).

### CasR and TGR5

CasR is Gαi/o- or Gαq-coupled, and its ligands are Ca2+, L-amino acids, and oligopeptides (157). NLRP3 inflammasome and associated IL-1β release are closely associated with inflammatory progression in RA (171, 172). Monocytes show increased CasR expression (173, 174). Monocytes and macrophages can phagocytose colloidal calmodulin particles to prevent extracellular calcification *in vivo*. In this process, increased extracellular Ca2+ concentrations can activate CasR, and thus activate the NLRP3 inflammasome in monocytes to promote IL-1β release for inflammation (175).

TGR5 is Gαs-coupled and its ligands are lithocholic acid, deoxycholic acid, chenodeoxycholic acid, and cholic acid (157). Bile acids mainly activate TGR5. TGR5 mRNA expression is reduced in PBMCs from RA patients and correlates with CRP and DAS28 levels. Lithocholic acid inhibits NF-kB activity and inflammation by binding TGR5 in PBMC, while it reduces proinflammatory factors in CIA mice, such as TNF-α, IL-6, IL-8, and IL-1β (176).

### Formyl Peptide Receptor-Like 1 (FPRL1)

FRP1 and FPRL1s are Gαi/o- or Gαq-coupled, and their ligands are N-formyl-methionine and N-formyl-metoligopeptides (157). Acute-phase serum amyloid A (A-SAA) and FPRL1 are expressed in RA FLSs, macrophages, and endothelial cells. A-SAA is associated with RA disease activity and is regulated by proinflammatory factors, which may be involved in the stromal degradation of RA through FPRL1-induced secretion of MMP-1 and MMP-3 from FLSs (177). In addition, A-SAA can promote FLS proliferation and angiogenesis by binding to FPRL1 (178). Similarly, annexin-1 may also induce MMP-1 secretion from FLS *via* FPRL1 (179).

### G Protein-Coupled Receptor Kinase 2 (GRK2)

GRK2 is a key enzyme involved in desensitization of many G proteins (180). Reduced T-cell expression of GRK2 promotes increased responses in CCL3, CCL4, and CCL5 (181). GRK2 in endothelial cells may regulate inflammatory responses by regulating Weibel-Palade body formation-mediated cytokinesis and histamine-stimulated aggregation of leukocytes to endothelial cells (180).

## Conclusion

The structure and signaling pathways of GPCRs have been investigated and explained in various diseases; however, their specific mechanisms and roles in RA remain to be elucidated. In this review, we discussed multiple GPCR receptors to clarify some of their roles and mechanisms. Nevertheless, many questions remain. First, chemokine receptors are currently the most intensively studied GPCRs in RA. There are two main aspects of RA drug strategies developed for chemokines and chemokine receptors: on one hand, corresponding ligands that can be selected to inhibit chemokine receptors and on the other, direct inhibition of chemokine receptors. Both options are currently investigated in several preclinical and clinical studies. However, the exact clinical efficacy still needs to be further observed in well-designed clinical trials. Unfortunately, some good preclinical trial results do not translate into an excellent clinical protocol. Some clinical trials on chemokines have faced difficulties, possibly owing to the widespread expression of chemokine receptors in a variety of cells, implying that a portion of chemokine receptors may be necessary for certain normal physiological processes, and therefore, potentially effective chemokine receptor inhibitors may specifically target receptor expression in certain pathogenic cell subpopulations. Second, the expression of GPCRs at different disease stages of RA may have diverse functional roles, such as CCR4. Clarifying the role of receptors at different stages is vital for drug development. Finally, the application of novel variable conformation modulators and biased agonists of GPCRs may be an essential tool for the future development of GPCR drugs for RA. So far, inhibition of single GPCR has failed to bring the desired outcomes. The combined inhibition of multiple GPCRs showed better efficacy. In addition, better use of modern techniques, such as synovial biopsy and arthroscopic surgery with the aid of computer imaging, may allow for better evaluation of the overall disease situation, and combined analysis using multi-omics and multiple molecular biology techniques may improve the current situation. Although the translation of preclinical GPCR results to clinical results for RA is still challenging, in-depth studies present a direction with a great potential.

## Author Contributions

JZ is responsible for the collection, collation, and writing of the original manuscript. KW, CC, PJ, LXX, LSX, and YS are accountable for the collection. SG and DH are responsible for the concept development, revision, and manuscript review. All authors reviewed and accepted the final version.

## Funding

This work was funded by the National Natural Science Funds of China (82074234 and 82071756), National Key Research and Development Project (2018YFC1705200 and 2018YFC1705203), Shanghai Chinese Medicine Development Office, National Administration of Traditional Chinese Medicine, Regional Chinese Medicine (Specialist) Diagnosis and Treatment Center Construction Project-Rheumatology, State Administration of Traditional Chinese Medicine, National TCM Evidence-Based Medicine Research and Construction Project, Basic TCM Evidence-Based Capacity Development Program, Shanghai Municipal Health Commission, and East China Region-based Chinese and Western Medicine Joint Disease Specialist Alliance.

## Conflict of Interest

The authors declare that the research was conducted in the absence of any commercial or financial relationships that could be construed as a potential conflict of interest.

## Publisher’s Note

All claims expressed in this article are solely those of the authors and do not necessarily represent those of their affiliated organizations, or those of the publisher, the editors and the reviewers. Any product that may be evaluated in this article, or claim that may be made by its manufacturer, is not guaranteed or endorsed by the publisher.

## Glossary

**Table:**

RA	rheumatoid arthritis
GPCRs	G protein-coupled receptors
NSAIDs	non-steroidal anti-inflammatory drugs
GDP	guanosine diphosphate
GTP	guanosine triphosphate
cAMP	cyclic adenosine monophosphate
PKC	protein kinase C
CCR	C-C motif chemokine receptor
CXCR	C-X-C motif chemokine receptors
XCR1	X-C motif chemokine receptor 1
CX3CR1	C-X3-C motif chemokine receptor 1
anti-CCP	anti-cyclic citrullinated peptide
DAS28	the disease activity score-28
RF	rheumatoid factor
FLSs	fibroblast-like synoviocytes
TNF&alpha	tumor necrosis factor &alpha
MMP	matrix metalloproteinase
CIA	the collagen-induced arthritis
IFN	interferon
CXCL10, RANKL	receptor activator of nuclear factor kappa-B ligand
CKLF1	chemokine-like factor 1
CRP	C-reactive protein
ESR	erythrocyte sedimentation rate
E2F2	E2F transcription factor 2
FOXP	forkhead box P
STAT5	signal transducer and activator of transcription 5
ROR	RAR-related orphan nuclear receptor
LPS	lipopolysaccharides
PGC1	proliferator-activated receptor gamma coactivator 1
ACPA	anti-cyclic citrullinated peptide antibody
ERK	extracellular signal-regulated kinase
VLA-4	very late activation antigen 4
VCAM-1	vascular cell adhesion molecule 1
SDF-1	stromal cell-derived factor 1
TLR	Toll-like receptor
NF-kB	nuclear factor kappa-light-chain-enhancer of activated B cells
Tfh	follicular helper T
P-gp	P-glycoprotein
Tfr	follicular regulatory T
ROS	reactive oxygen species
Bregs	regulatory B cells
JNK	Janus kinase
MAPK	mitogen-activated protein kinase
MNCs	mononuclear cells
MC	melanocortin
NOS2	nitric oxide synthase 2
a-MSH	alpha-Melanocyte-stimulating hormone
sTNFr	soluble tumor necrosis factor receptor
PUFA	polyunsaturated fatty acids
TAK1	transforming growth factor-&beta activated kinase 1
HMG-1	high mobility group protein 1
IL	interleukin
MCP-1	monocyte chemoattractant protein 1
ICAM-1	intercellular adhesion molecule 1
VCAM-1	vascular cellular adhesion molecule 1
VEGF	vascular endothelial growth factor
PBMC	peripheral blood mononuclear cell
KKS	kallikrein-kinin system
pKal	prekallikrein
FXII	factor XII
HK	high-MW kininogen
sRANKL	soluble receptor activator of nuclear factor kappa B ligand
PI3Ks	phosphoinositide 3-kinases
PKB	protein kinase B
FPRL1	formyl peptide receptor-like 1
A-SAA	acute-phase serum amyloid A
GRK2	G protein-coupled receptor kinase 2
